# Lysine-specific demethylase 5C promotes hepatocellular carcinoma cell invasion through inhibition BMP7 expression

**DOI:** 10.1186/s12885-015-1798-4

**Published:** 2015-10-26

**Authors:** Xuening Ji, Shi Jin, Xiaotong Qu, Kejun Li, Hongjiang Wang, Hui He, Fuchao Guo, Lei Dong

**Affiliations:** Department of Oncology, Zhongshan Hospital of Dalian University, No. 6 Jiefang Street, Zhongshan District, Dalian, 116001 China; Department of Laparoscopic Surgery, First Affiliated Hospital of Dalian Medical University, No.193, Lianhe Street, Shahekou District, Dalian, 116001 China; Department of Second Neurology, The Frist Affiliated Hospital of Dalian Medical University, No.222, Zhongshan Street, Xigang District, Dalian, 116000 China; Department of Breast Surgery, First Affiliated Hospital of Dalian Medical University, No.193, Lianhe Street, Shahekou District, Dalian, 116001 China; Department of general surgery, The first people’s Hospital of jinzhou District in Dalian City, No.683, Stalin Road, Jinzhou District, Dalian, 116100 China

**Keywords:** KDM5C, BMP7, HCC, Metastasis

## Abstract

**Background:**

Hepatocellular carcinoma (HCC) is the most common type of tumor and is associated with high morbidity and mortality rates. Patients with HCC routinely undergo surgery followed by adjuvant radiation therapy and chemotherapy. Despite such aggressive treatment approaches, median survival times remain under 1 year in most cases. KDM5C is a member of the family of JmjC domain-containing proteins that removes methyl residues from methylated lysine 4 on histone H3 lysine 4 (H3K4). KDM5C has been proposed as an oncogene in many types of tumors; however, its role and underlying mechanisms in HCC remain unclear.

**Methods:**

Expression level of KDM5C was examined by RT-PCR, and IHC. Forced expression of KDM5C was mediated by retroviruses, and KDM5C was downregulated by shRNAs expressing lentiviruses. Migration and invasion of HCC cells was measured by wound healing, Transwell and Matrigel assays respectively.

**Results:**

In this study, we report that KDM5C is abundantly expressed in invasive human HCC cells. Cellular depletion of KDM5C by shRNA inhibited HCC cell migration, invasion and epithelial-mesenchymal transition *in vitro*, and markedly decreased the metastasis capacity of invasive HCC cells in the liver and lung. Furthermore, ectopic expression of KDM5C in HCC cells promoted cell migration, invasion and epithelial-mesenchymal transition via the inactivation of BMP7. Knockdown of BMP7 significantly promotes shKDM5C-induced cell migration inhibition.

**Conclusions:**

Taken together, these data suggest that KDM5C-mediated BMP7 inactivation is essential for HCC cell invasion.

**Electronic supplementary material:**

The online version of this article (doi:10.1186/s12885-015-1798-4) contains supplementary material, which is available to authorized users.

## Background

Hepatocellular carcinoma (HCC) is a highly aggressive tumor characterized by its lack of response to conventional chemo-, radio-, and immunotherapies [1]. HCC cells extensively invade normal tissues, which contribute to the continued poor prognosis for these tumors by preventing complete surgical resection [2, 3]. Invading tumor cells are resistant to conventional therapies and invasiveness is enhanced by antiangiogenic approaches [4, 5]. Thus, an effective strategy to prevent invasion of HCC cells into surrounding normal tissues is needed.

Lysine (K)-specific demethylase 5C (KDM5C) is a member of the family of JmjC domain-containing proteins that specifically removes methyl residues from tri-, di-, and monomethylated lysine 4 on histone H3 lysine 4 (H3K4) that are associated with active genes [6]. KDM5C is a transcriptional repressor that harbors intrinsic histone demethylase activity [7]. Trimethylation at H3K4 is an important histone mark associated with actively transcribed genes, and KDM5C specifically demethylates H3K4me3 to a transcriptionally inactive state [8]. KDM5C is up-regulated in multiple tumor cell lines [9–11]. Furthermore, KDM5C promotes proliferation of cancer cells, and knockdown of KDM5C causes a significant delay in the G1/S transition [12, 13]. KDM5C appears to promote tumorigenesis through the specific repression of antiproliferative genes [14, 15]. Thus, KDM5C may act as an oncogene, but whether KDM5C plays a role in HCC metastasis remains unknown.

Bone morphogenetic proteins (BMPs) signal via specific serine/threonine kinase receptors on the cell surface known as bone morphogenetic protein receptors (BMPRs) [16]. Both type-I and -II BMPRs are required for signal transduction. The type-I receptor BMPRIA preferentially binds ligands of the Dpp class, such as BMP2 and BMP4, whereas the type-II receptor BMPRIB binds ligands of the 60A class, such as BMP7 [16–18]. After ligand binding, the type-I/type-II receptor complex phosphorylates Smad1, 5 and 8 proteins that translocate to the nucleus to regulate target gene expression [19]. Previous studies have shown that BMP-7 exert anti-invasive actions by inhibiting TGF-beta-induced expression of integrin beta [20, 21].

In the current study, we show that KDM5C overexpression predicts poor prognosis in HCC patients undergoing curative resection. Additionally, we present the evidence that KDM5C expression promotes HCC cells invasion, metastasis and EMT. These functional effects of KDM5C were exerted through control of BMP7 transcriptional expression via H3K4me3. The down-regulation of BMP7 triggered by KDM5C therefore enforces HCC cells oncogenesis and metastasis. Our findings provide a novel mechanistic role of KDM5C in HCC metastasis, suggesting that KDM5C may serve as a potential therapeutic target for advanced HCCs.

## Methods

### Chemicals and antibodies

Lipofectamine 2000 transfection and TRIZOL LS Reagents were purchased from Invitrogen (Grand Island, NY, USA). Antibodies against KDM5C, Histone H3, H3K4me1 (monomethylated K4), H3K4me2 (dimethylated K4), H3K4me3 (trimethylated K4), H3K27me3 (trimethylated K27) were purchased from Abcam (Cambridge, MA, USA). E-cadherin, N-cadherin, vimentin, BMP7, and β-actin antibodies were from Cell Signaling technology (Danvers, MA, USA). Anti-α-catenin antibody was from BD (Franklin Lakes, NJ, USA).

### Patients and specimens

Eighteen tumor and para-cancerous tissues which, were used for qRT-PCR analysis, were randomly collected from HCC patients who underwent curative resection with informed consent between 2013 and 2014 at the department of laparoscopic surgery, First Affiliated Hospital of Dalian Medical University. All tissues were collected immediately upon resection of the tumors in the operation theater, transported in liquid nitrogen, and then stored at −80 °C. Another 221 hepatocellular carcinoma tissues which, were used for immunohistochemical analysis, were randomly collected from HCC patients who underwent curative resection with informed consent between 2008 and 2014 at the Department of Hepatobiliary Surgery, First Affiliated Hospital of Dalian Medical University. Tumor staging was based on the 6th edition of the tumor-node-metastasis (TNM) classification of the International Union Against Cancer. The clinicopathologic characteristics of the 221 hepatocellular carcinoma tissues are summarized in Table 1. Follow-up data were summarized at the end of March 2015, with a median observation time of 62.4 months. Study protocols were approved by the Hospital Ethics Committee of Dalian Medical University, and written informed consent was obtained from patients based on the Declaration of Helsinki.

**Table 1:** KDM5C staining and clinicopathologic characteristics of 221 hepatocellular carcinoma patients

Variables	KDM5C staining		Total	P*
	Low	High		
Age (y)				0.513
≤ 50	61 (73 %)	22 (27 %)	83	
> 50	99 (72 %)	39 (28 %)	138	
Sex				0.405
Male	133 (73 %)	49 (27 %)	182	
Female	31 (79 %)	8 (21 %)	39	
HBsAg				0.072
Negative	46 (80 %)	11 (20 %)	57	
Positive	115 (60 %)	49 (40 %)	164	
HCV				0.169
Negative	139 (66 %)	72 (34 %)	211	
Positive	8 (80 %)	2 (20 %)	10	
AFP				0.534
≤ 20	73 (75 %)	25 (25 %)	98	
> 20	91 (74 %)	32 (26 %)	123	
γ-GT(U/L)				0.187
≤ 54	72 (67 %)	36 (33 %)	108	
> 54	81 (72 %)	32 (28 %)	113	
Liver cirrhosis				0.566
No	29 (69 %)	13 (31 %)	42	
Yes	122 (68 %)	57 (32 %)	179	
Tumor diameter (cm)				0.028
≤ 5	133 (68 %)	62 (32 %)	195	
> 5	12 (46 %)	14 (54 %)	26	
Microvascular invasion				<0.001
Absence	74 (80 %)	19 (20 %)	93	
Present	53 (41 %)	75 (59 %)	128	
Tumor encapsulation				0.096
Complete	78 (74 %)	27 (26 %)	105	
None	77 (66 %)	39 (34 %)	116	
Tumor differentiation				<0.001
I + II	115 (73 %)	42 (27 %)	157	
III + IV	26 (41 %)	38 (59 %)	64	
TNM stage				0.139
I	121 (64 %)	68 (36 %)	189	
II + III	23 (72 %)	9 (28 %)	32	

*HBsAg* hepatitis B surface antigen, *AFP* α-fetoprotein, *γ-GT* γ-glutamyl transferase, *TNM* tumor-nodesmetastasis

*p-value < 0.05 was considered statistically significant. *p*-values were calculated using the Pearson chi-square test

### Histological and immunohistochemical analysis

The normal human liver tissues, human tumor tissues, and lungs dissected from mice were fixed in 4 % paraformaldehyde in phosphate-buffered saline (PBS) overnight and subsequently embedded in paraffin wax. Sections cut at a thickness of 4 μm were stained with hematoxylin and eosin for histological analysis. Immunohistochemical analysis was performed for different markers in these arrays as described previously. The proportion of stained cells (lower, <30 % staining; higher, ≥30 % staining) was semiquantitatively determined following published protocols [22, 23].

### Cell culture

HCC cells (ATCC, Manassas, VA, USA) were cultured under the following conditions: Huh7, and MHCC97L cell lines were cultured using 10 % fetal bovine serum (Cat#10099-141, Invitrogen, Carlsbad, CA) in either RPMI-1640 (Cat#C11875, Invitrogen). L02 cell lines were cultured using 10 % fetal bovine serum (Invitrogen) in Dulbecco’s modified Eagle medium (Cat#C11965, Invitrogen). Cell culture was according to manufacturer’s protocol. All the cell lines were grown at 37 °C in a 5 % CO_2_/95 % air atmosphere and were revived every 3 to 4 months.

### Establishment of KDM5C and BMP7 stable expression and KDM5C and BMP7 knockdown cell lines

Retroviral construct containing human pBabe-*KDM5C* cDNA, pcDNA3.1-*BMP7* cDNA and pSuper.retro.puro with shRNA against human *KDM5C* and si*BMP7* were prepared as described previously [22, 24]. The generation of retrovirus supernatants and transfection of hepatocellular carcinoma cells were conducted as described previously [22, 25]. The expression of KDM5C and BMP7 was confirmed by qRT-PCR and Western blotting analysis.

### Wound healing assay

Cells were seeded in 6 cm culture plates, and the cell monolayers were wounded by scratching with sterile plastic 200 μl micropipette tips and photographed using phase-contrast microscopy. The migration distance of each cell was measured after the photographs were converted to Photoshop files.

### Cell invasion and motility assay

Invasion of cells was measured in Matrigel (BD, Franklin Lakes, NJ, USA) -coated Transwell inserts (6.5 mm, Costar, Manassas, VA, USA) containing polycarbonate filters with 8-μm pores as detailed previously. The inserts were coated with 50 μl of 1 mg/ml Matrigel matrix according to the manufacturer’s recommendations. 2 × 10^5^ cells in 200 μl of serum-free medium were plated in the upper chamber, whereas 600 μl of medium with 10 % fatal bovine serum were added to lower well. After 24 h incubation, cells that migrated to the lower surface of the membrane were fixed and stained. For each membrane, five random fields were counted at × 10 magnification. Motility assays were similar to Matrigel invasion assay except that the Transwell insert was not coated with Matrigel.

### Western blotting

Cells were lysed in lysis buffer and total protein contents were determined by the Bradford method. 30 μg of lysis were separated by reducing SDS-PAGE and probed with specific antibodies. Blots were washed and probed with respective secondary peroxidase-conjugated antibodies, and the bands visualized by chemoluminescence (Amersham Biosciences).

### qRT-PCR

Total RNA was extracted using Trizol reagent and cDNA was synthesized using SuperScript II Reverse Transcriptase (Invitrogen). qRT-PCR and data collection were performed with an ABI PRISM 7900HT sequence detection system. The primers used for the amplification of the indicated genes are available in Additional file 1: Table S1.

### Gene expression profiling

Total RNA quality and quantity were determined using Agilent 2100 Bioanalyzer and NanoDrop ND-1000. Affymetrix HU U133 plus 2.0 arrays were used according to manufacturer’s protocol. The data were initially normalized by robust multiarray average (RMA) normalization algorithms in expression console software (Affymetrix). Significantly altered genes between KDM5C overexpression and its control cells were considered by scatter plots and the genes up- and down-regulated ≥5-fold. Clustering analysis was done using gene list by Gene Cluster v3.0 software, and heat maps were visualized using Java TreeView v1.1.4r3 software. Gene set enrichment analysis was carried out using ConceptGen (http://conceptgen.ncibi.org/core/conceptGen/index.jsp). Gene sets were either obtained from the ConceptGen or from published gene signatures.

### Chromatin immunoprecipitation (ChIP)-qPCR

Chromatin Immunoprecipitation kit (Cat. 17–371) was purchased from Millipore and ChIP experiments were carried out essentially as described [26]. Immnuoprecipitated DNA was analyzed on the ABI PRISM 7900HT sequence detection system. The primers used for detection of promoters after ChIP are available upon request.

### In vivo tumor metastasis assays

Nude mice were purchased from the Shanghai Slac Laboratory Animal Co. Ltd and maintained in microisolator cages. All animals were used in accordance with institutional guidelines and the current experiments were approved by the the Dalian Medical University Experimental Animal Care Commission. For metastasis assays, cells were resuspended in PBS at a concentration of 1×10^7^ cells ml^−1^. Cell suspension (0.1 ml) was injected into tail veins of nude mice. All of the mice were killed by CO_2_ 60 days after inoculation.

### Statistical analysis

Results were analyzed with SPSS13.0 statistical software. Correlation between KDM5C expression and clinicopathologic parameters was evaluated using the Chi-square (*χ*2) test, and quantitative variables were analyzed by the independent *t* test. The survival probability was estimated by Kaplan-Meier method, and the comparison of survival curves between groups was done with the log-rank test. The statistical significance of the differences between mean values was determined by *P* < 0.05.

## Results

### KDM5C is highly expressed and correlated with distant metastasis in HCC

**Fig. 1:**
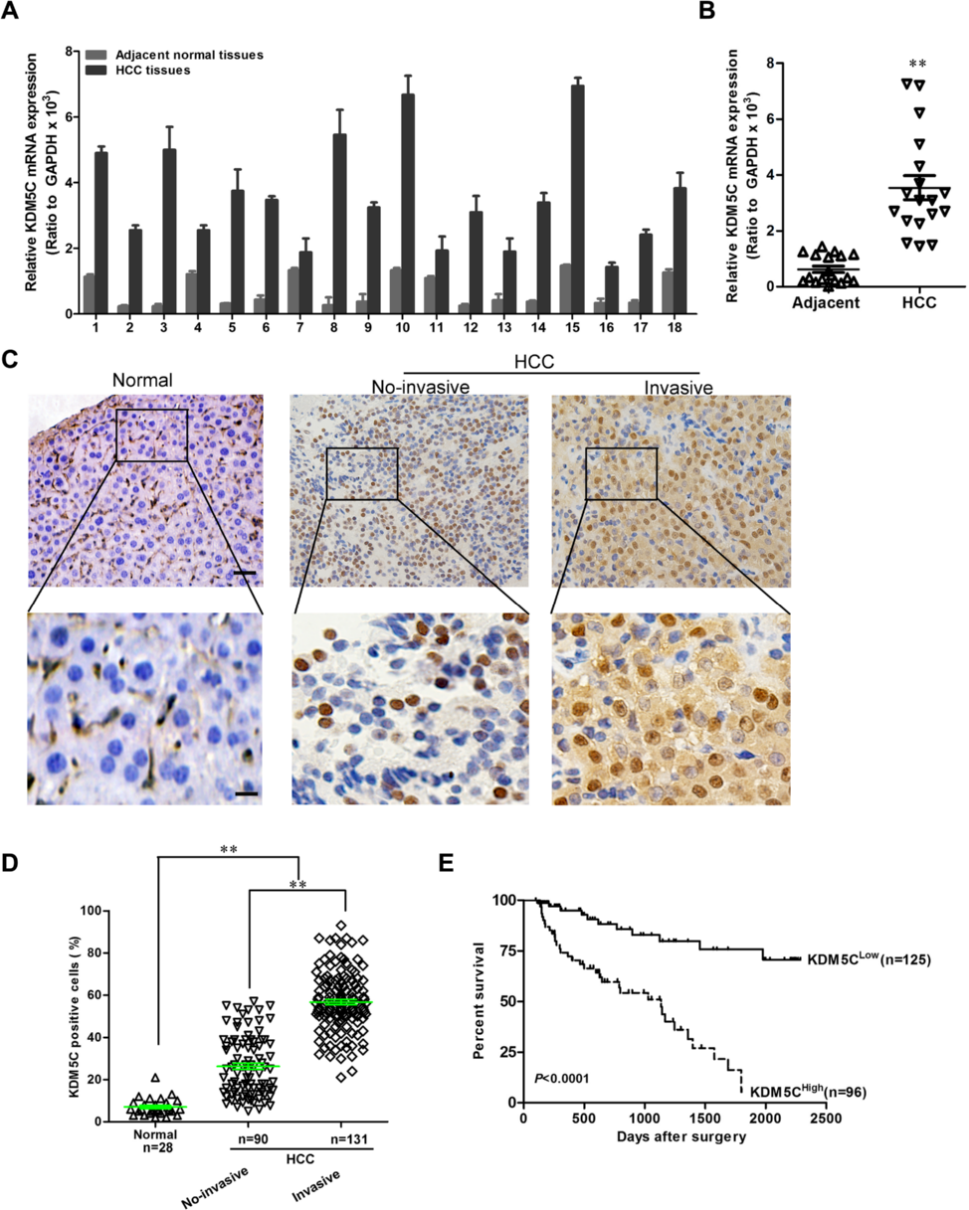
KDM5C is highly expressed in HCC. **a** KDM5C mRNA expression was analyzed by quantitative RT-PCR in tumors and adjacent tissues. **b** Comparison of the expression levels of KDM5C mRNA in adjacent normal tissues, and HCCs. **c** KDM5C protein expression was analyzed by immunohistochemical analysis in 221 cases HCC tissues and the representative results were shown. **d** semiquantification of KDM5C expression in normal tissues, no-invasive HCC and invasive HCC tissues. **e** The association between KDM5C expression in HCC and the survival time of selected patients was analyzed with Kaplan-Meier survival analysis. Scale bars, 50 μm (upper) and 20 μm (lower) in C. **, *P* < 0.01 is based on the Student *t* test. All results are from three independent experiments. Error bars, SD

To investigate whether KDM5C might be involved in HCC, the mRNA expression level of KDM5C in HCC tissues and its matched normal adjacent liver tissues was determined by qRT-PCR in 18 samples. As compared with normal liver tissues, HCC specimens showed overexpression of KDM5C (Fig. 1a and b). We then analyzed KDM5C protein expression in more HCC samples by IHC. We observed that the level of KDM5C positive cells was markedly higher in HCC tissues than the level in the normal liver tissues (Fig. 1c). Most importantly, KDM5C overexpression was consistently significantly correlated to distant metastasis in these HCC samples (Fig. 1c and d). To investigate the relationship between KDM5C expression and clinicopathological parameters in the 221 cases with HCCs, these cases were first divided into two subgroups: “low KDM5C expression” and “high KDM5C expression” as defined in the immunohistochemistry section of “Materials and methods”. Significant correlations were found between KDM5C expression and tumor diameter, microvascular invasion, and tumor differentiation. There were no statistical connections between KDM5C expression and the rest clinicopathological parameters, such as patient age, gender, and HBsAg (Table 1). The association between KDM5C expression in HCC and the survival time of selected patients was analyzed with Kaplan-Meier survival analysis (Fig. 1e). The median overall survival time of high KDM5C expression group was significantly shorter than that of low KDM5C expression group (*P* < 0.001). These results collectively indicate a functional role of KDM5C in aggressive behaviors of HCCs.

### Transfection efficiency of KDM5C in HCC cell lines

**Fig. 2:**
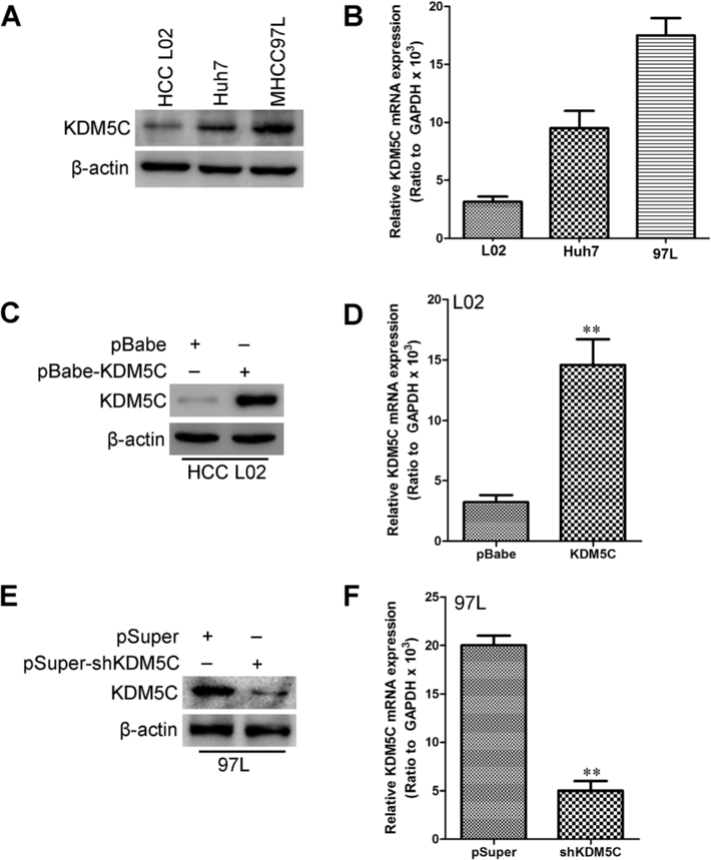
Transfection efficiency of KDM5C in HCC cell lines. **a** Expression of KDM5C protein was measured by Western blotting in L02, Huh7 and MHCC97L cell lines. **b** expression of KDM5C mRNA was measured by qRT-PCR in L02, Huh7 and MHCC97L cell lines. **c** The transfection efficiency of KDM5C in L02 cells was analyzed by western blot. **d** The transfection efficiency of KDM5C in L02 cells was analyzed by qRT-PCR. **e** The transfection efficiency of KDM5C shRNA or control vector in MHCC97L cells were analyzed by western blot. **f** the transfection efficiency of KDM5C shRNA or control vector in MHCC97L cells were analyzed by qRT-PCR. **, *P* < 0.01 is based on the Student *t* test. All results are from three independent experiments. Error bars, SD

We next analyzed the expression of KDM5C in the immortalized normal human liver cell line, L02, and HCC cell lines Huh7 and MHCC97L. Significantly higher expression of KDM5C protein (Fig. 2a) and mRNA (Fig. 2b) was evident in Huh7 and MHCC97L cell lines than in immortalized normal human liver cell line L02. The KDM5C expression plasmid pBabe-KDM5C was transfected into 02 cells. After selection with puromycin, KDM5C expression was assayed by western blot (Fig. 2c) and qRT-PCR (Fig. 2d). MHCC97L is a highly invasive HCC cell line commonly used in HCC research. This cell line was infected with pSuper-shKDM5C or the control (pSuper) to investigate the effect of KDM5C knockdown on the migration and invasion of HCC cells. Western blot (Fig. 2e), and qRT-PCR (Fig. 2f) demonstrated that the protein and mRNA expression level of KDM5C was significantly suppressed in the cells infected with pSuper-shKDM5C.

### KDM5C promotes migratory and invasive capacities of HCC cells in vitro

**Fig. 3:**
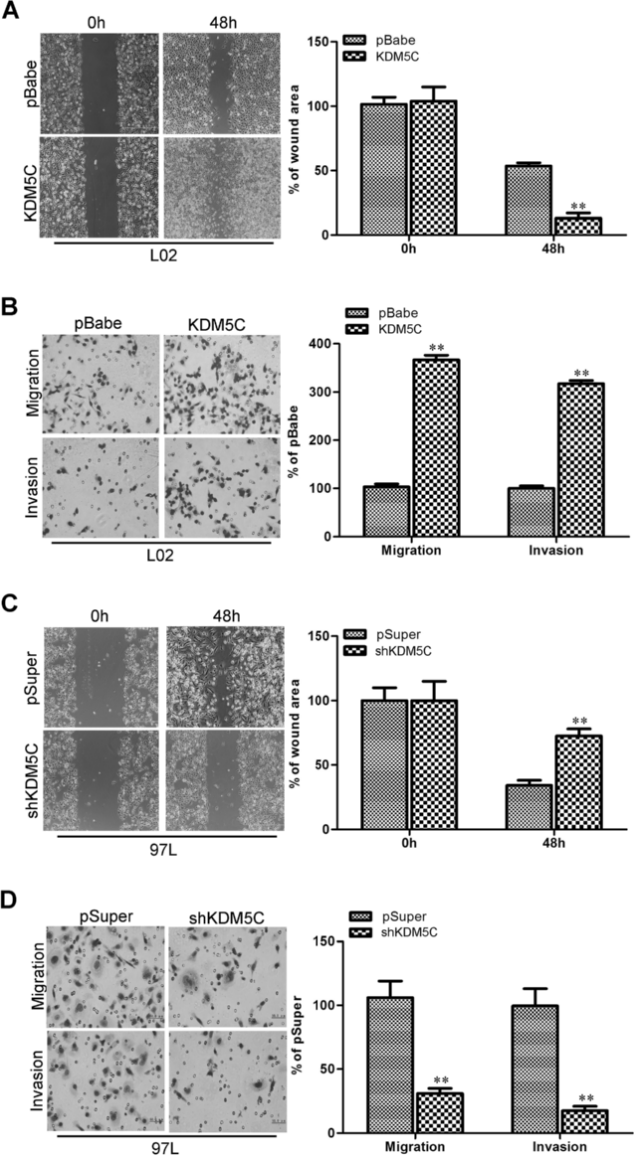
KDM5C promotes migratory and invasive capacities of HCC cells in vitro. **a** L02-pBabe-KDM5C and its control vector cells were subjected to wound healing assays; the uncovered areas in the wound healing assays were quantified as a percentage of the original wound area. **b** L02-pBabe-KDM5C and its control vector cells were subjected to Transwell migration (top), and Matrigel invasion assays (bottom), quantification of migrated cells through the membrane and invaded cells through Matrigel of each cell line are shown as proportions of their vector controls. **c** MHCC97L-pSuper-shKDM5C and its control vector cells were subjected to wound healing assays, the uncovered areas in the wound healing assays were quantified as a percentage of the original wound area. **d** MHCC97L-pSuper-shKDM5C and its control vector cells were subjected to Transwell migration (top), and Matrigel invasion assays (bottom), quantification of migrated cells through the membrane and invaded cells through Matrigel of each cell line are shown as proportions of their vector controls. **, *P* < 0.01 is based on the Student *t* test. All results are from three independent experiments. Error bars, SD

The effect of KDM5C on HCC cell migration was first assessed by wound healing assay. L02-pBabe-KDM5C cells had significantly faster closure of the wound area compared to control cells (Fig. 3a). This result was confirmed by Boyden’s chamber assay (Fig. 3b). Moreover, L02-pBabe-KDM5C cells showed a greater degree of invasion through Matrigel (Fig. 3b). In contrast, silencing KDM5C dramatically reduced the migratory and invasive capacity of MHCC97L cells (Fig. 3c and d). These results indicate that KDM5C promotes migratory and invasive behaviors in HCC cells in vitro.

### KDM5C promotes HCC cells metastasis in vivo

**Fig. 4:**
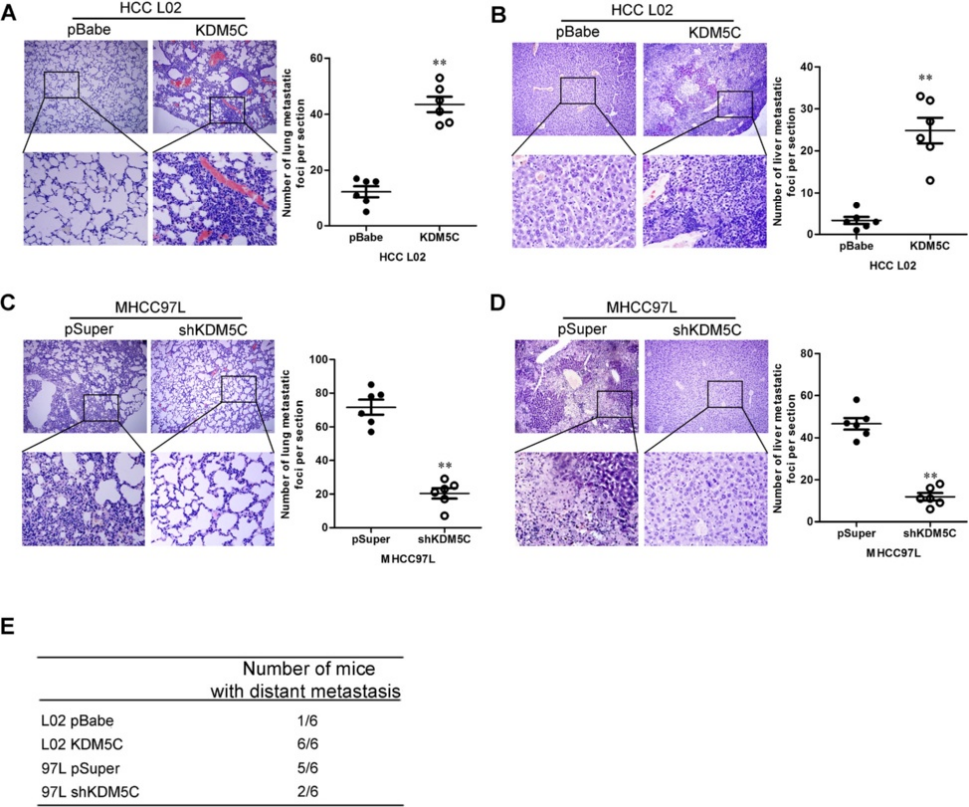
KDM5C promotes HCC cells metastasis in vivo. a The numbers of metastatic foci per section in lung of individual mouse with injection of L02-pBabe-KDM5C or its control cells. **b** The numbers of metastatic foci per section in liver of individual mouse with injection of L02-pBabe-KDM5C or its control cells. **c** The numbers of metastatic foci per section in lung of individual mouse with injection of MHCC97L-pSuper-shKDM5C or its control cells. **d** The numbers of metastatic foci per section in liver of individual mouse with injection of MHCC97L-pSuper-shKDM5C or its control cells. **e** The total numbers of mice with distant metastasis at 60 days after injection of L02-pBabe-KDM5C, MHCC97L-pSuper-shKDM5C or their control cells. *n* = 6 for subcutaneous transplantation and *n* = 5 for tail vein injection. **, *P* < 0.01 is based on the Student *t* test. All results are from three independent experiments. Error bars, SD

We then investigated the functional relevance of KDM5C for metastasis *in vivo*. L02-pBabe-KDM5C, MHCC97L-pSuper-shKDM5C and their corresponding control cells were injected into nude mice through the tail vein. KDM5C overexpression not only significantly increased the number of mice with distant metastasis (Fig. 4e), but also dramatically increased the number of metastatic tumors in both lung and liver of each mouse (Fig. 4a and b). Silencing KDM5C in MHCC97L cells inhibited metastatic behavior, both in terms of the number of mice with distant metastasis (Fig. 4e) and the number of metastatic tumors in the lung and liver of each mouse (Fig. 4c and d). Therefore, the *in vivo* results further demonstrate the critical role of KDM5C in HCC cells metastasis.

### KDM5C regulates the transition between epithelial and mesenchymal phenotypes in HCC cells

**Fig. 5:**
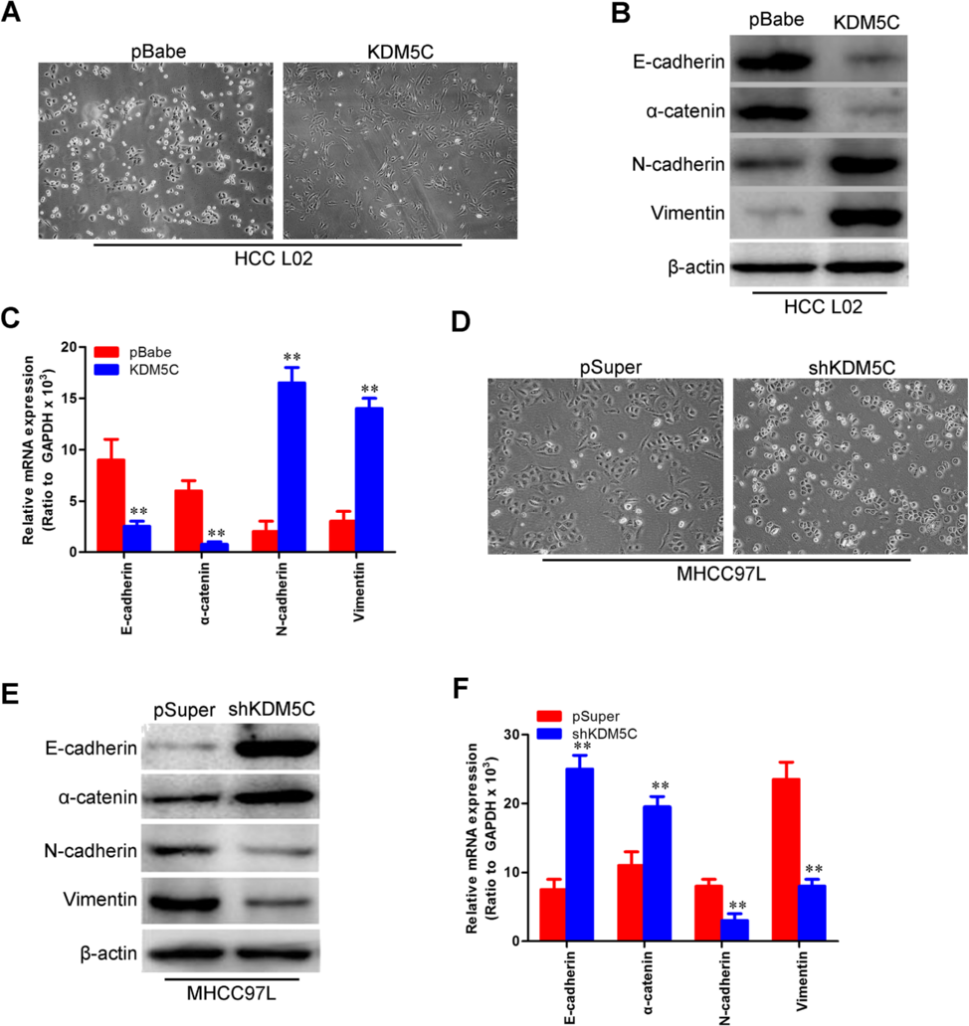
KDM5C regulates the transition between epithelial and mesenchymal phenotypes in HCC cells. **a** Representative phase-contrast images of L02 cells showed KDM5C overexpression-modulated morphologic changes. **b** expression of epithelial and mesenchymal marker was analyzed by Western blotting in L02-pBabe-KDM5C and its control cells. **c** Expression of epithelial and mesenchymal marker was analyzed by qRT-PCR in L02-pBabe-KDM5C and its control cells. **d** Representative phase-contrast images of MHCC97L cells showed KDM5C knockdown-modulated morphologic changes. **e** Expression of epithelial and mesenchymal marker was analyzed by Western blotting in MHCC97L-pSuper-shKDM5C and its control cells. **f** expression of epithelial and mesenchymal marker was analyzed by qRT-PCR in MHCC97L-pSuper-shKDM5C and its control cells. **, *P* < 0.01 is based on the Student *t* test. All results are from three independent experiments. Error bars, SD

We then observed the cells morphological changes and found that L02-pBabe-KDM5C cells exhibited fibroblastic morphology (Fig. 5a). This observation was further confirmed by expression analyses of epithelial and mesenchymal markers. We showed that KDM5C overexpression decreased the levels of epithelial markers (E-cadherin and α-catenin) and increased the levels of mesenchymal markers (N-cadherin and vimentin) in cell lines (Fig. 5b and c). Conversely, MHCC97L-pSuper-shKDM5C cells reverted to an epithelial phenotype as compared to their respective control cells (Fig. 5d). Consistent with this, silencing KDM5C increased levels of epithelial markers, and decreased levels of mesenchymal markers (Fig. 5e and f). Taken together, these findings suggest that KDM5C plays an important role in regulating EMT-MET plasticity of HCC cells.

### KDM5C regulates BMP7 expression through H3K4 trimethylation

**Fig. 6:**
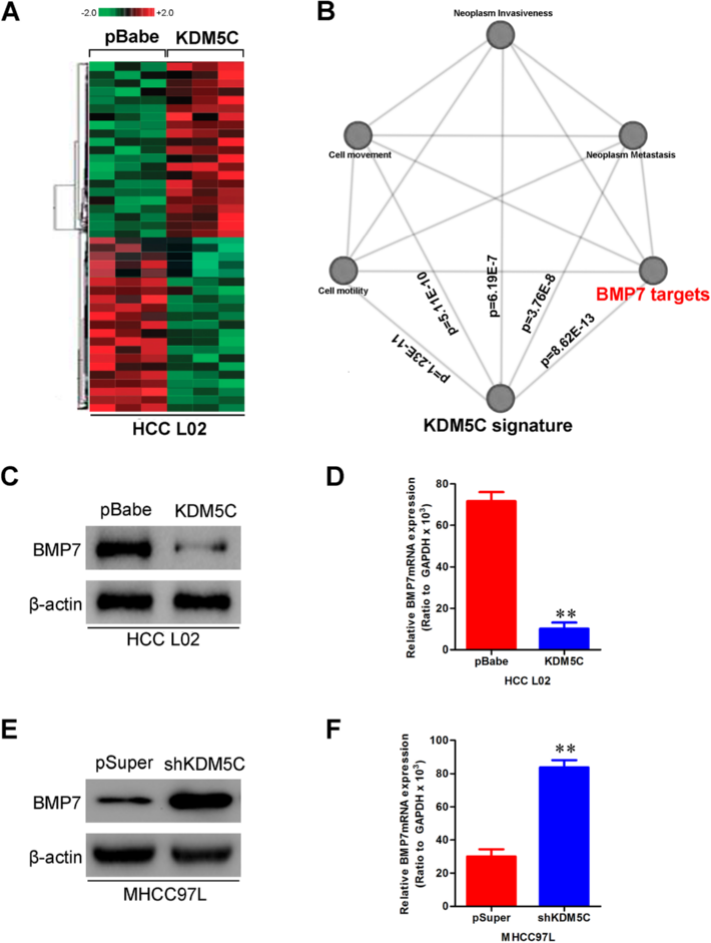
KDM5C downregulates BMP7 expression in HCC cells. **a** Supervised hierarchical clustering of the genes differentially expressed after KDM5C overexpression in L02 cells. **b** Gene set enrichment analysis was carried out using ConceptGen. **c** Protein level of BMP7 was measured by Western blotting assay in L02-pBabe-KDM5C and its control cells. **d**, mRNA level of BMP7 was measured by qRT-PCR assay in L02-pBabe-KDM5C and its control cells. **e** Protein level of BMP7 was measured by Western blotting assay in MHCC97L-pSuper-shKDM5C and its control cells. **f** mRNA level of BMP7 was measured by qRT-PCR assay in MHCC97L-pSuper-shKDM5C and its control cells. **, *P* < 0.01 is based on the Student *t* test. All results are from three independent experiments. Error bars, SD

To better understand the mechanisms by which KDM5C engaged in HCC development and progression, we performed gene expression profiling on L02-pBabe-KDM5C and its control cells. Microarray analyses identified a list of genes significantly differentially expressed after KDM5C overexpression including downregulation of *BMP7* (Fig. 6a). Furthermore, gene set enrichment analysis indicated that proliferation, neoplasm metastasis and invasion, cell movement and motility, and BMP7 related gene signatures were significantly changed in KDM5C overexpression cells (Fig. 6b), supporting the idea that KDM5C regulates proliferation, EMT and cancer invasion and metastasis. These data also led us to hypothesize that KDM5C exerts these functions possibly via BMP7. To test this, we first determined whether BMP7 is a downstream target of KDM5C in HCC cells. Expression of BMP7 in the cells with altered KDM5C expression was further evaluated by Western blotting and qRT-PCR. L02-pBabe-KDM5C cells exhibited greatly decreased both BMP7 protein and mRNA levels (Fig. 6c and d), whereas silencing KDM5C in MHCC97L cells dramatically increased its mRNA and protein levels (Fig. 6e and f).

**Fig. 7:**
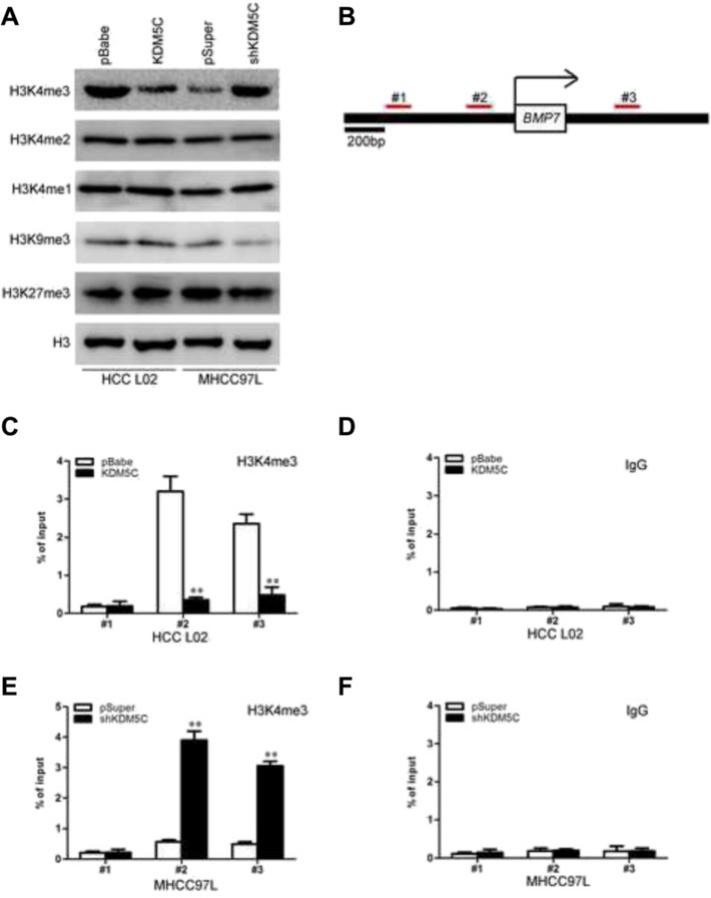
KDM5C regulates BMP7 expression through H3K4 trimethylation. **a** The abundance of H3 lysine methylation was assessed in HCC cells with KDM5C overexpression or shRNA by Western blotting using whole-cell lysate; total H3 and β-actin were used as a loading control. **b** Schematic presentation of three regions relative to the BMP7 transcriptional start site used as primers to test histone occupied abundance. **c** and **d** qChIP was performed to assess H3K4me3 occupancy in L02-pBabe-KDM5C and its control cells. IgG was used as negative control. **e** and **f**, qChIP was performed to assess H3K4me3 occupancy in MHCC97L-pSuper-shKDM5C and its control cells. IgG was used as negative control. “Percentage of input” indicates the ratio of DNA fragment of each promoter region bound by H3K4me3 to the total amount of input DNA fragment without H3K4me3 antibody pull-down. **, *P* < 0.01 is based on the Student *t* test. All results are from three independent experiments. Error bars, SD

**Fig. 8:**
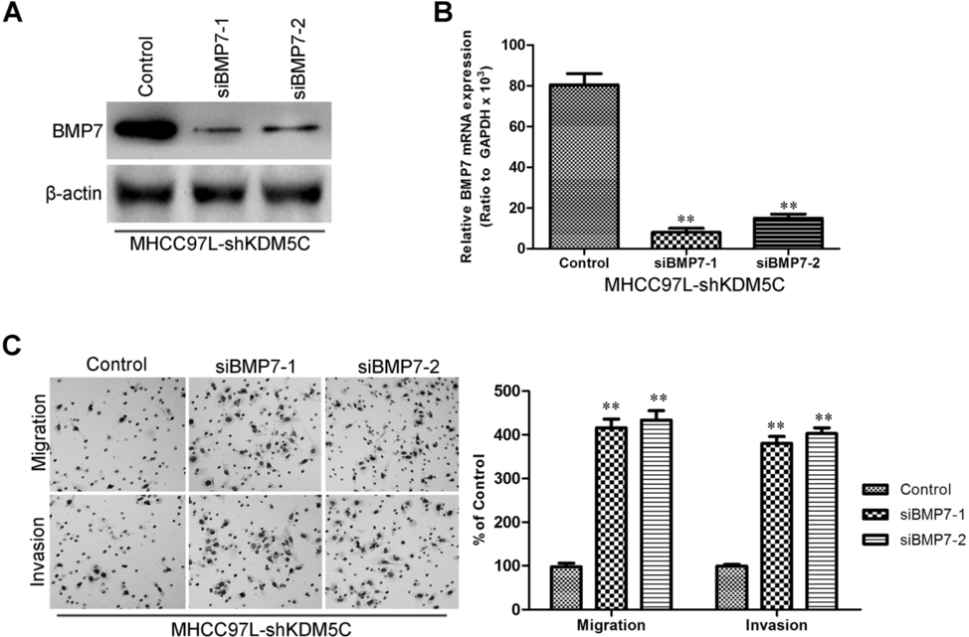
BMP7 mediates KDM5C-induced migration and invasion in HCC cells. **a** Silenced BMP7 protein expression in MHCC97L-pSuper-shKDM5C cells was measured by Western blotting. **b**, Silenced BMP7 mRNA expression in MHCC97L-pSuper-shKDM5C cells was measured by qRT-PCR. **c** MHCC97L-pSuper-shKDM5C cells treated with siBMP7 were subjected to Transwell migration (top), and Matrigel invasion assays (bottom), quantification of migrated cells through the membrane and invaded cells through Matrigel of each cell line are shown as proportions of their vector controls. **, *P* < 0.01 is based on the Student *t* test. All results are from three independent experiments. Error bars, SD

We then explored how KDM5C regulates BMP7 expression at the transcriptional level. KDM5C complexes are frequently involved in chromatin regulation [27, 28]. To determine whether KDM5C regulates specific histone modifications in HCC cells, histone modification patterns were measured after modulation of KDM5C expression. Among histone H3K4 and H3K27, we found that only H3K4 was affected by KDM5C. Ectopic expression of KDM5C decreased H3K4me1, H3K4me2, and H3K4me3 while silencing of KDM5C increased these modifications (Fig. 7a). Because H3K4me3 is associated with active transcription, we tested whether KDM5C expression was correlated with the H3K4me3 modification at the *BMP7* gene promoter in HCC cells (Fig. 7b). Quantitative chromatin immunoprecipitation (qChIP) assays were performed in L02-pBabe-KDM5C and its corresponding control cells. We found that KDM5C expression was associated with decreased H3K4me3 levels at region −548 to −316 bp (Fig. 7B2) and +682 to +891 bp (Fig. 7B2) of the *BMP7* promoter in L-02-pBabe-KDM5C cells (Fig. 7c and d). More occupancy of those *BMP7* gene promoter regions by H3K4me3 was detected in MHCC97L-pSuper-shKDM5C cells (Fig. 7e and f). The occupancy of chromatin repressors such as H3K27me3 at the *BMP7* gene promoter was not changed by altered CUL4A expression (Additional file 2: Figure S1). These results clearly indicate that KDM5C induces transcriptional inactivation of BMP7 through regulating H3K4me3 and decreasing H3K4me3 to the *BMP7* gene promoter. On the basis of the indispensable role of BMP7 in the biologic functions of KDM5C, we silenced BMP7 in MHCC97L-pSuper-shKDM5C cells (Fig. 8a and b). Of note, silencing BMP7 in MHCC97L-pSuper-shKDM5C cells significantly increased the migration and invasion (Fig. 8c). Taken together, these results show that BMP7 mediates KDM5C-induced migration and invasion in hepatocellular carcinoma cells.

## Discussion

Hepatocellular carcinoma (HCC) is the fifth most common malignancy in the world and the second leading cause of cancer death in Asia [1]. The high mortality rate of HCC is caused by frequent tumor metastasis, postsurgical recurrence, and late detection at advanced stages [5]. HCC is associated with multiple risk factors and is now recognized as a both genetic and epigenetic disease [29, 30]. While the sequential accumulation of various genetic changes in hepatocarcinogenesis has been extensively studied, the contribution of epigenetic alterations to HCC development and progression has remained relatively poorly understood. Histone methylation is a dynamic epigenetic process that participates in a diverse array of cellular processes and has been found to associate with cancer, including HCC [13]. Recently, several histone demethylases have been identified that catalyze the removal of methylation from histone H3 lysine residues [13, 31]. Previous studies had found that KDM5C, also named JARID1C, as a histone demethylase that can catalyze the removal of all three methyl groups from the H3K4 lysine residue [7]. However, its role and underlying mechanisms in HCCs still unknown.

To our knowledge, this is the first research to show that KDM5C plays an important role in HCC. We found that as compared with normal tissues, HCC specimens showed overexpression of KDM5C and its overexpression was significantly correlated with distant metastasis in HCC tissues. High expression of KDM5C was associated with decreased overall survival of HCC patients. KDM5C overexpression in HCC cells induced EMT, migration, invasion *in vitro* and enhanced metastatic capacities *in vivo*. In contrast, silencing KDM5C reversed these events in invasive HCC cells. We also showed that a mechanistic link between KDM5C and BMP7 through KDM5C-mediated regulation of H3K4m3, which subsequently leads to transcriptional downregulation of BMP7 expression. These results lead us to propose a model for KDM5C regulation of EMT and metastasis through transcriptional regulation of BMP7 in HCCs.

Histone lysine demethylases (KDMs) are epigenetic enzymes that can remove both repressive and activating histone marks [32]. KDM5 family members are capable of removing the histone H3 lysine 4 dimethylation-activating marks, rendering them potential players in the down-regulation of tumor suppressors and suggesting that their activity could repress oncogenes [32]. Histone demethylase KDM5C is required for proper DNA replication at early origins [33]. KDM5C dictates the assembly of the pre-initiation complex, driving the binding to chromatin of the pre-initiation proteins, through the demethylation of the histone mark H3K4me3 [14]. The putative role of KDM5C as an oncogene in cancer development is supported by the observations that KDM5C is highly expressed in prostate cancer and other malignant tumors relative to normal tissues [9]. Consistent with this research, in the present study we showed that overexpression of KDM5C promoted HCC cells proliferation and enhanced tumor formation in vivo. Interestingly, our study points to a novel function of KDM5C in HCC metastasis through regulating EMT.

HCC cells with ectopic expression of KDM5C displayed an EMT phenotype, including the associated stimulatory effects on migration and invasion *in vitro*. Interestingly, our results indicate that KDM5C not only promotes EMT, but silencing of KDM5C also leads to MET. All of these characteristics induced by KDM5C *in vitro* culminated to increased numbers of distant metastases *in vivo*. These empirical findings provide a mechanistic framework to explain the clinical observations that HCC patients with high levels of KDM5C in tissue samples have more chance of distant metastasis, a significantly shorter overall and disease-free survival.

Bone morphogenetic proteins (BMPs) are secreted signaling molecules belonging to the transforming growth factor (TGF)-beta superfamily [17]. Recent studies demonstrated that the expression patterns of BMP7 are altered in several tumors. BMP7 is essential during development, and more recently has also been implicated in cancer pathogenesis [20]. The roles of several transcription factors as EMT regulators have been extensively reported [22]. In our effort to elucidate the mechanism how KDM5C modulates EMT in HCC cells, we identified BMP7 as an effective mediator of KDM5C-induced these phenomena. BMP7 is one of the most frequently mutated tumor suppressors in human cancer including HCCs [34]. BMP7 also suppresses migration, genetic deletion of the BMP7 tumor suppressor gene promotes cell motility, and BMP7 reconstitution or overexpression inhibits cell motility in some cell types [20, 35]. Mechanistically, BMP7 reduces cell motility through a variety of pathways, and decreased ERK and AKT are two important targets of BMP7 [36]. The mechanistic connection between KDM5C and BMP7 was previously unknown. In this study, we showed that modulation of KDM5C expression altered the methylation status of H3K4 at the BMP7 gene promoter, which in turn transcriptionally controlled expression of BMP7. However, we did not detect any influence of KDM5C expression on the methylation status of H3K27. Thus, we conclude that KDM5C transcriptionally inactivates BMP7 expression through H3K4 demethylation and reduces H3K4me3 to the BMP7 gene promoter, and consequently resulting in increase in migration and invasion both in vitro and in vivo.

## Conclusions

In conclusion, we found that KDM5C expression was generally higher in HCC lesions compared with non-tumor tissues. Our in vitro and in vivo data demonstrate that KDM5C has a vital function in promoting cell mobility, which is partially by the regulation of BMP7 expression. Thus, we propose that the candidate tumor oncogene KDM5C may be an effective novel therapeutic target in the management of HCC.

## Additional files

Additional file 1: Table S1. The primers used for the amplification of the indicated genes.(DOCX 17 kb) Additional file 2: Figure S1. A and B, qChIP was performed to assess H3K27me3 occupancy in HCC L02-pBabe-KDM5C and its control cells. IgG was used as negative control. C and D, qChIP was performed to assess H3K27me3 occupancy in MHCC97L-pSuper-shKDM5C and its control cells. IgG was used as negative control. “Percentage of input” indicates the ratio of DNA fragment of each promoter region bound by H3K4me3 to the total amount of input DNA fragment without H3K4me3 antibody pull-down. (JPEG 1102 kb)
