# Supplementary material for: Lysine-specific demethylase 5C promotes hepatocellular carcinoma cell invasion through inhibition BMP7 expression
Source: BMC Cancer. 2015 Oct 26;15:801. doi: 10.1186/s12885-015-1798-4 (PMC4624178; doi:10.1186/s12885-015-1798-4)
Supplement: Additional file 1: Table S1. — The primers used for the amplification of the indicated genes.(DOCX 17 kb) [file 12885_2015_1798_MOESM1_ESM.docx]

Supplemental Table 1

| Primer | Sequence(5’ to 3’) | Applications |
| --- | --- | --- |
| GAPDH-S370 | GCT GGC GCT GAG TAC GTC GT |  |
| GAPDH-AS821 | ACG TTG GCA GTG GGG ACA CG | *GAPDH* RT-PCR |
| KDM5C-S287 | CCGAGGCTGTCAACTTTTGC |  |
| KDM5C-AS858 | GTCAAAGGACTCAGCCCGAA | *KDM5C* RT-PCR |
| E-cadherin-S1117  E-cadherin-AS1562  N-cadherin-S1152  N-cadherin-AS1562  Vimentin-S83  Vimentin-AS518  α-catenin-S961  α-catenin-AS1168 | TGG GCT GGA CCG AGA GAG TTT C  ATC CAG CAC ATC CAC GGT GAC G  CCG GTT TCA TTT GAG GGC ACA TGC  GCC GTG GCT GTG TTT GAA AGG C  AAC TTA GGG GCG CTC TTG TC  GGT GGA CGT AGT CAC GTA GC  TCA TTG TGG ACC CCT TGA GC  TTA CGT CCA GCA TTG CCC AT | *E-cadherin* RT-PCR  *N-cadherin* RT-PCR  *Vimentin* RT-PCR  *α-catenin* RT-PCR |
