# Supplementary figures and images for: Lysine-specific demethylase 5C promotes hepatocellular carcinoma cell invasion through inhibition BMP7 expression
Source: BMC Cancer. 2015 Oct 26;15:801. doi: 10.1186/s12885-015-1798-4 (PMC4624178; doi:10.1186/s12885-015-1798-4)

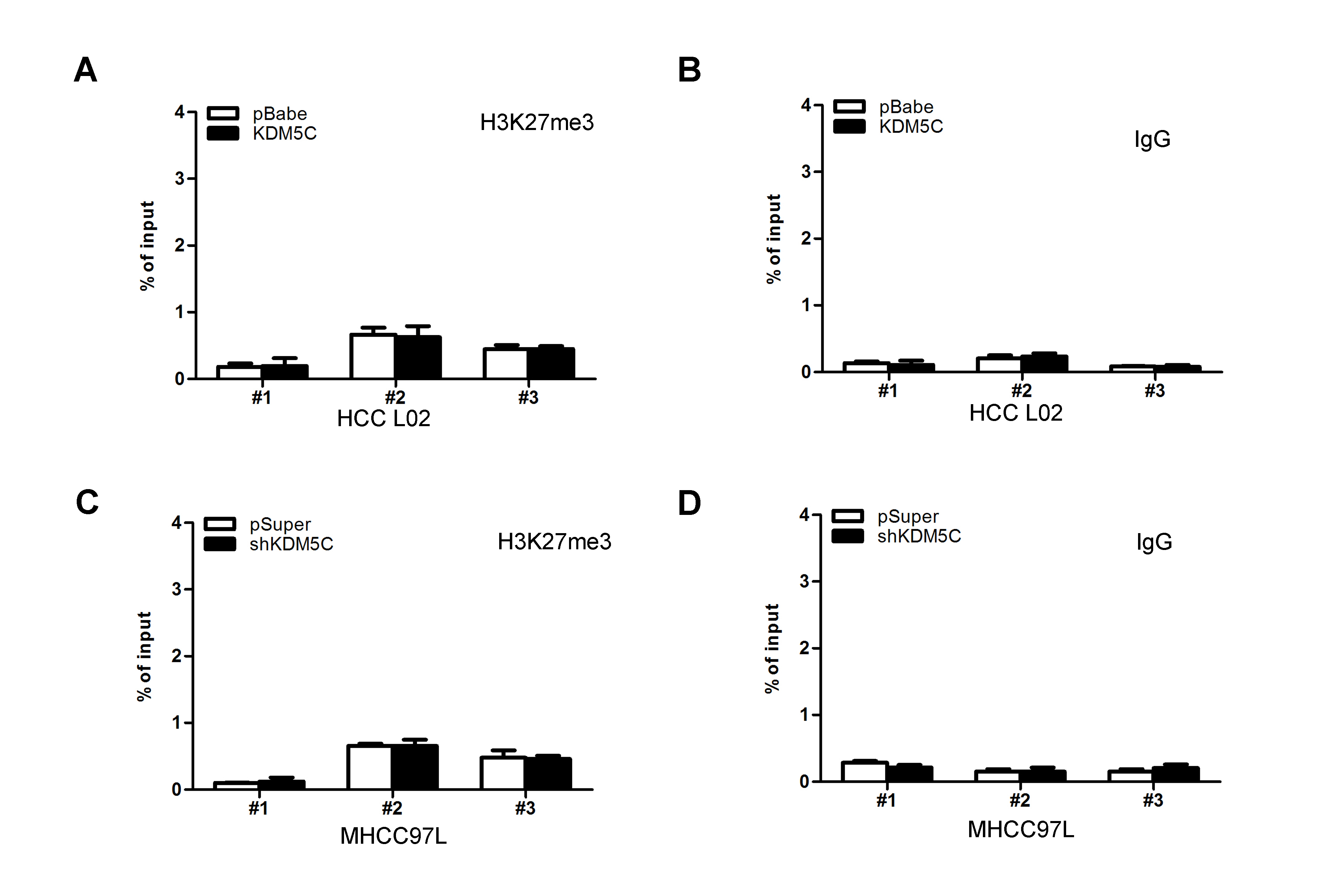

Supplement: Additional file 2: Figure S1. — A and B, qChIP was performed to assess H3K27me3 occupancy in HCC L02-pBabe-KDM5C and its control cells. IgG was used as negative control. C and D, qChIP was performed to assess H3K27me3 occupancy in MHCC97L-pSuper-shKDM5C and its control cells. IgG was used as negative control. “Percentage of input” indicates the ratio of DNA fragment of each promoter region bound by H3K4me3 to the total amount of input DNA fragment without H3K4me3 antibody pull-down. (JPEG 1102 kb) [file 12885_2015_1798_MOESM2_ESM.jpg]
